# The International IgA Nephropathy Network Prediction Tool Underestimates Disease Progression in Indian Patients

**DOI:** 10.1016/j.ekir.2022.03.016

**Published:** 2022-03-24

**Authors:** Soumita Bagchi, Ashish Datt Upadhyay, Adarsh Barwad, Geetika Singh, Arunkumar Subbiah, Raj Kanwar Yadav, Sandeep Mahajan, Dipankar Bhowmik, Sanjay Kumar Agarwal

**Affiliations:** 1 Department of Nephrology, All India Institute of Medical Sciences, New Delhi, India; 2 Department of Biostatistics, All India Institute of Medical Sciences, New Delhi, India; 3 Department of Pathology, All India Institute of Medical Sciences, New Delhi, India

**Keywords:** IgA nephropathy, Indians, outcome, prediction equation, risk, validity

## Abstract

**Introduction:**

International IgA nephropathy (IgAN) network (IIgANN) prediction tool was developed to predict risk of progression in IgAN. We attempted to externally validate this tool in an Indian cohort because the original study did not include Indian patients.

**Methods:**

Adult patients with primary IgAN were stratified to low, intermediate, higher, and highest risk groups, as per the original model. Primary outcome was reduction in estimated glomerular filtration rate (eGFR) by >50% or kidney failure. Both models were evaluated using discrimination: concordance statistics (C-statistics), time-dependent receiver operating characteristic (ROC) curves, R^2^d, Kaplan–Meier survival curves between risk groups and calibration plots. Reclassification with net reclassification improvement and integrated discrimination improvement (IDI) was used to compare the 2 models with and without race.

**Results:**

A total of 316 patients with median follow-up of 2.8 years had 87 primary outcome events. Both models with and without race showed reasonable discrimination (C-statistics 0.845 for both models, R^2^d 49.9% and 44.7%, respectively, and well-separated survival curves) but underestimated risk of progression across all risk groups. The calibration slopes were 1.234 (95% CI: 0.973–1.494) and 1.211 (95% CI: 0.954–1.468), respectively. Both models demonstrated poor calibration for predicting risk at 2.8 and 5 years. There was limited improvement in risk reclassification risk at 5 and 2.8 years when comparing model with and without race.

**Conclusion:**

IIgANN prediction tool showed reasonable discrimination of risk in Indian patients but underestimated the trajectory of disease progression across all risk groups.

## Graphical abstract

**Figure:**
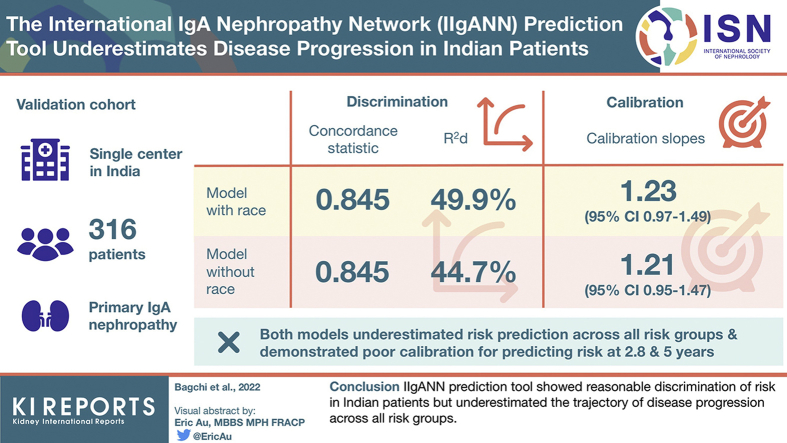

IgAN is the most common primary glomerular disease worldwide.^1^ Race/ethnicity is recognized as a risk factor for disease severity and progression.^2^^,^^3^ Ethnic/racial disparities have been reported which suggest that the disease is more aggressive in south Asians.^4^^,^^5^ The clinical presentation ranges from incidentally detected urinary abnormalities, such as microscopic hematuria, subnephrotic proteinuria, and nephrotic-range proteinuria, to rapidly progressive glomerulonephritis with significant variability in disease course and renal survival.^1^ Risk stratification is important to counsel patients and guide treatment and monitoring strategies. Multiple prediction models have been developed based on clinical and histologic criteria but^6^, ^7^, ^8^, ^9^, ^10^ have not been used widely in clinical practice because they were not validated across ethnicities or did not use the widely accepted Oxford MEST histologic classification system.^11^ The recent IIgANN prediction tool was developed and tested in a large multiethnic population and integrates both clinical characteristics and the Oxford MEST criteria.^12^ The derivation and the validation cohorts did not include patients of South Asian ethnicity. Because they have a higher risk of rapid deterioration of renal function,^5^ we aimed to assess the performance of this model in an Indian cohort.

## Methods

We conducted a single-center retrospective cohort study to evaluate the validity of the IIgANN prediction tool in an independent cohort of Indian patients. Medical records (outpatient and inpatient files and biopsy reports) of adult (≥18 years) patients diagnosed with biopsy-proven primary IgAN between January 2013 and March 2020 were analyzed. Patients with <6 months of follow-up were excluded unless they had progressed to the primary outcome in <6 months. We also excluded patients who had permanent kidney failure at the time of kidney biopsy (eGFR < 15 ml/min per 1.73 m^2^); had secondary causes of IgAN such as chronic liver disease, Henoch-Schönlein purpura; had a second coexisting disease on kidney biopsy such as diabetic nephropathy, a systemic disease-like diabetes or malignancy which may affect kidney function; or if MEST score was not available.

The study was approved by the Institute Ethics Committee, and the requirement for informed consent was waived. Results have been presented according to the TRIPOD guidelines for the validation of risk prediction models.^13^

### Sample Size

**Figure 1:**
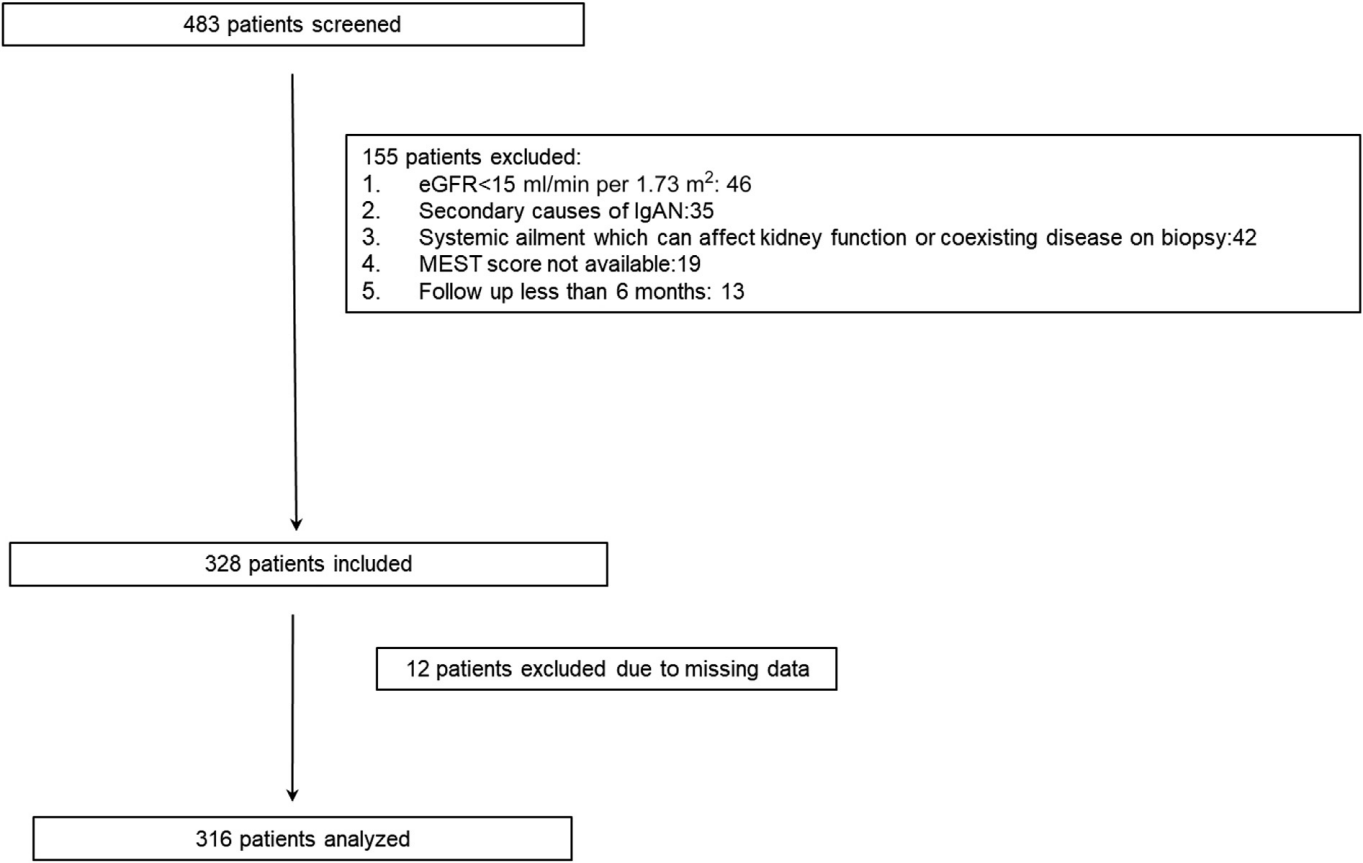
Flowchart of patients screened, recruited, and included in final analysis. eGFR, estimated glomerular filtration rate; IgAN, IgA nephropathy.

There were 328 patients eligible to be included in this study. Nevertheless, 12 patients (3.7%) had missing data and were excluded from the study, and a total of 316 patients were included in the final analysis. Figure 1 shows the flowchart for the cohort selection.

### Predictors and Outcome

To calculate the linear predictor (lp) and prediction probability of the primary outcome for each patient, we used both models with and without race proposed by the original study.^12^ The predictors used in both models, defined and retrieved according to the original study were as follows: age, eGFR, mean arterial pressure, proteinuria, prior use of renin-angiotensin-aldosterone system blockers and immunosuppression at the time of kidney biopsy. eGFR was calculated using the Chronic Kidney Disease Epidemiology Collaboration equation. Data for proteinuria were obtained from a 24-hour urine protein collection or a spot urine protein creatinine ratio as available and expressed as gram per day. Mean arterial pressure was calculated as the sum of diastolic pressure and one third of the pulse pressure. The kidney biopsy results were evaluated, and MEST score was assigned by the renal pathologists. For the model with race which requires additional information on race (Chinese, Japanese, White, or Other), we classified our patients as “Other.”

The time to origin was the date of kidney biopsy. Primary outcome was defined as sustained reduction in eGFR by >50% or kidney failure (eGFR < 15 ml/min per 1.73 m^2^, requiring maintenance dialysis or undergone renal transplantation).

### Statistical Analyses

Data were analyzed using STATA 14.0 software (StataCorp, College Station, TX and R version 4.0.5, R Foundation for Statistical Computing, Vienna, Austria). Median and interquartile ranges were calculated for continuous variables, and categorical variables were presented as numbers and percentages. We stratified our patients into the following 4 risk groups as per the centile of the linear predictors: low risk (<16th), intermediate risk (16th–50th), higher risk (50th–84th), and highest risk (>84th).

There were 328 patients eligible after the inclusion and exclusion criteria. Nevertheless, 12 patients had missing data and were excluded from the study, and a total of 316 patients were included in the final analysis.

A cox regression model was fitted for the primary outcome with the linear predictor as the only variable. The hazard ratios were calculated keeping the lowest risk group as a reference group.

The performance of the proposed model was evaluated using discrimination, calibration, and reclassification. Discrimination was evaluated using C-statistic: Harrell c index, Gonen and Heller’s K C-statistics, R^2^d, and time-dependent ROC curves.^14^ The area under the curve of the ROC curves was calculated. We calculated the calibration slope with slope value >1 indicating greater discrimination. Continuous net reclassification index and IDI were used to compare the prediction models with and without race to estimate the reclassification of the clinical risk. Net reclassification improvement and integrated discrimination improvement (IDI) with 95% CIs not containing 0 were considered significant with a value > 0 suggesting positive improvement and a value < 1 indicating negative improvement. Kaplan–Meier survival analysis with log-rank test was done to compare the predicted and observed outcomes within risk groups. For calibration, the observed and predicted risks of the primary outcome were compared during the follow-up period among the risk groups according to the linear predictor. Calibration was also shown using plots with predicted versus observed risks of primary outcome by tenths of the predicted risk. Predicted risk was the mean predicted risk overall and in each group, whereas observed risk was obtained using the Kaplan–Meier method. Because our cohort had a median follow-up of 2.8 years, we evaluated the ROC curves and predicted versus observed risks at 5 years and 2.8 years.

## Results

The characteristics of our patients and the original derivation and validation cohorts are shown in Table 1. The median age was similar in all the 3 cohorts, but we had higher proportion of males in our study. Our patients had higher proteinuria compared with the original cohorts (median proteinuria 2.6 g/d vs. 1.2 and 1.3 g/d, respectively). They had lower median eGFR compared with the original cohorts (56.2 ml/min per 1.73 m^2^ vs. 83 and 90 ml/min per 1.73 m^2^, respectively). On evaluating the kidney biopsy specimens, we observed a higher prevalence of M1 lesions (77.9% vs. 38% and 42%, respectively), less E1 lesions (9.2% vs. 17% and 42%, respectively), and marginally higher T1/T2 lesions (39.0% vs. 30% and 29%, respectively). In addition, 16.5% of our patients had crescents on biopsy. Prior use of renin-angiotensin-aldosterone blockers was similar in all 3 groups (27.9% vs. 32% and 30%, respectively) whereas our patients had slightly higher prior immunosuppression exposure (14.2% vs. 9% and 7%, respectively). Primary outcome was observed in 87 6% (27.5%) patients in our study, of which 45 had progressed to end-stage renal failure. Primary outcome events were noted in 18% and 19% of the patients in original derivation and validation cohorts, respectively. Our median follow-up was 2.8 years, which was shorter than that of the original cohorts.

**Table 1:** Comparison of the clinical and histologic characteristics of our cohort with the original derivation and validation cohorts

Characteristics	Indian cohort (*n =* 316)	Original derivation cohort (*n =* 2781)	Original validation cohort (*n =* 1146)
Age (yr) (median, IQR)	31.5 (25-40.5)	36 (28–45)	35 (27-45)
Males (%)	223 (70.6)	1608 (57.80)	565 (49.3)
MAP (mm Hg) (median, IQR)	98.7 (96.3–106.7)	97 (89–106)	93 (85–103)
Proteinuria at biopsy (g/d) (median, IQR)	2.6 (1.5–4.0)	1.2 (0.7–2.2)	1.3 (0.6–2.4)
eGFR (ml/min per 1.73 m^2^) (median, IQR)	56.2 (38.2–90.8)	83 (57–108)	90 (65–113)
eGFR category, *n* (%)			
<30 ml/min per 1.73 m^2^	42(13.3)	142 (5)	37 (3)
30–60 ml/min per 1.73 m^2^	122 (38.6)	657 (24)	191 (17)
60–90 ml/min per 1.73 m^2^	70 (22.2)	800 (29)	350 (30)
>90 ml/min per 1.73 m^2^	82 (26.0)	1182 (42)	568 (50)
MEST lesions, *n* (%)			
M1	246 (77.9)	1054 (38)	481 (42)
E1	29 (9.2)	478 (17)	476 (42)
S1	216 (68.4)	2137 (77)	912 (80)
T1	95 (30.1)	686 (25)	207 (18)
T2	28(8.9)	128(5)	122 (11)
C1	42(13.3)		
C2	10(3.2)		
RAAS blocker use at or before biopsy, *n* (%)	88 (27.9)	862 (32)	320 (30)
Immunosuppression use at or before biopsy, *n* (%)	45 (14.2)	252 (9)	81 (7)
Duration of follow-up, yr (median, IQR)	2.8 (1.7–4.2)	4.8 (3.0–7.6)	5.8 (3.4–8.5)
Primary outcome observed, *n* (%)	87 (27.5)	492 (18)	213 (19)

eGFR, estimated glomerular filtration rate; IQR, interquartile range; MAP, mean arterial pressure; RAAS: renin-angiotensin-aldosterone.

### Performance of the IIgANN Prediction Tool

**Figure 2:**
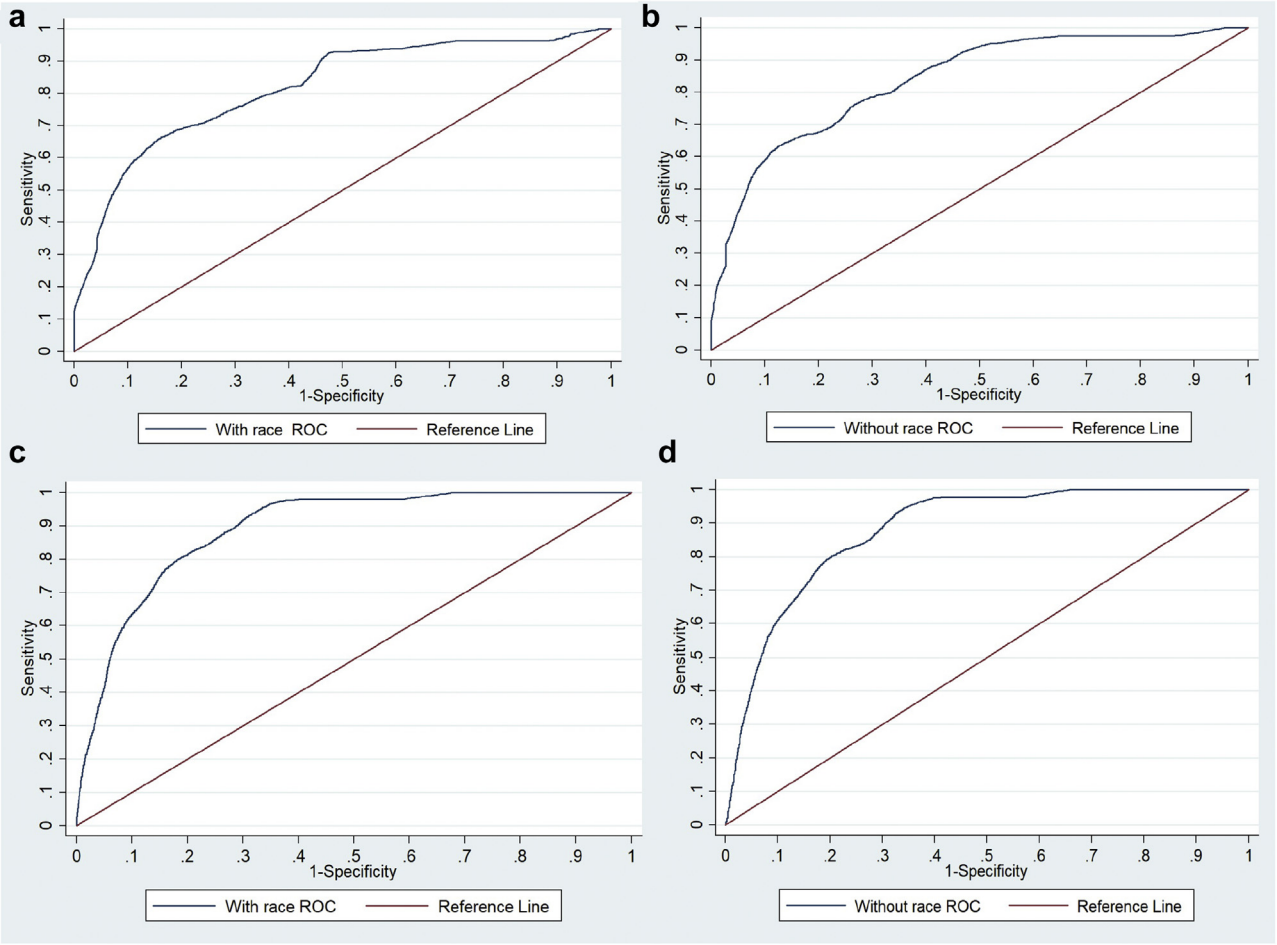
AUC of receiver operating curve analysis at 5 years for (a) full model with race and (b) full model without race and at 2.8 years for (c) full model with race and (d) full model without race. AUC, area under the curve; ROC, receiver operating characteristic.

Both the full models showed good discrimination in our cohort (Table 2). The Harrell c index was 0.845 (95% CI: 0.810–0.879) for the full model with race and 0.845 (95% CI: 0.811–0.879) for the full model without race. Gonen and Heller’s K C-statistic was 0.787 for the full model with race and 0.777 for the full model without race. The area under the ROC curve values at 5 years (Figure 2a and b) using the 2 models with and without race were 0.838 and 0.819 and at 2.8 years (Figure 2c and d) were 0.881 and 0.891, respectively. The overall calibration slopes (Table 2) were 1.2 (95% CI: 0.97–1.49) and 1.2 (95% CI: 0.95–1.46), respectively. The R^2^d for the full model with and without race was 49.9% and 44.7%, respectively.

**Table 2:** Concordance statistics, calibration slopes, NRIs, and IDIs of full models in our cohort

Variables	Full model with race	Full model without race
Harrell c index	0.845 (95% CI: 0.810–0.879)	0.845 (95% CI: 0.811–0.879)
AUC at 5 yr	0.838	0.819
AUC at 2.8 yr	0.881	0.891
Calibration slope	1.234 (95% CI: 0.973–1.494)	1.211 (95% CI: 0.954–1.468)
Calibration at 5 yr	1.211	1.211
Calibration at 2.8 yr	1.234	1.211
5-year performance compared with the full model without the race		
NRI	0.222(95% CI: 0.058–0.383)	
NRI (events)	0.444 (95% CI: 0.326–0.567)	
NRI (nonevents)	−0.222 (95% CI: −0.295 to −0.144)	
IDI	0.010 (95% CI: −0.005 to 0.029)	
2.8-year performance compared with the full model without the race		
NRI	0.347 (95% CI: 0.230–0.482)	
NRI (events)	0.451 (95% CI: 0.324–0.579)	
NRI (nonevents)	−0.105 (95% CI: −0.144 to −0.068)	
IDI	0.021 (95% CI: 0.008–0.035)	

AUC, area under the curve; IDI, integrated discrimination improvement; NRI, net reclassification improvement.

Compared with the full model without race, the full model with race (where race was designated as “other” for our cohort) showed limited improvement in risk reclassification for predicting 5-year risk, with net reclassification improvement of 0.222 (95% CI: 0.058–0.383) and IDI of 0.010 (95% CI: −0.005 to 0.029), which increased marginally at 2.8 years with 0.347 (95% CI: 0.230–0.482) with IDI of 0.021 (95% CI: 0.008–0.035).

**Figure 3:**
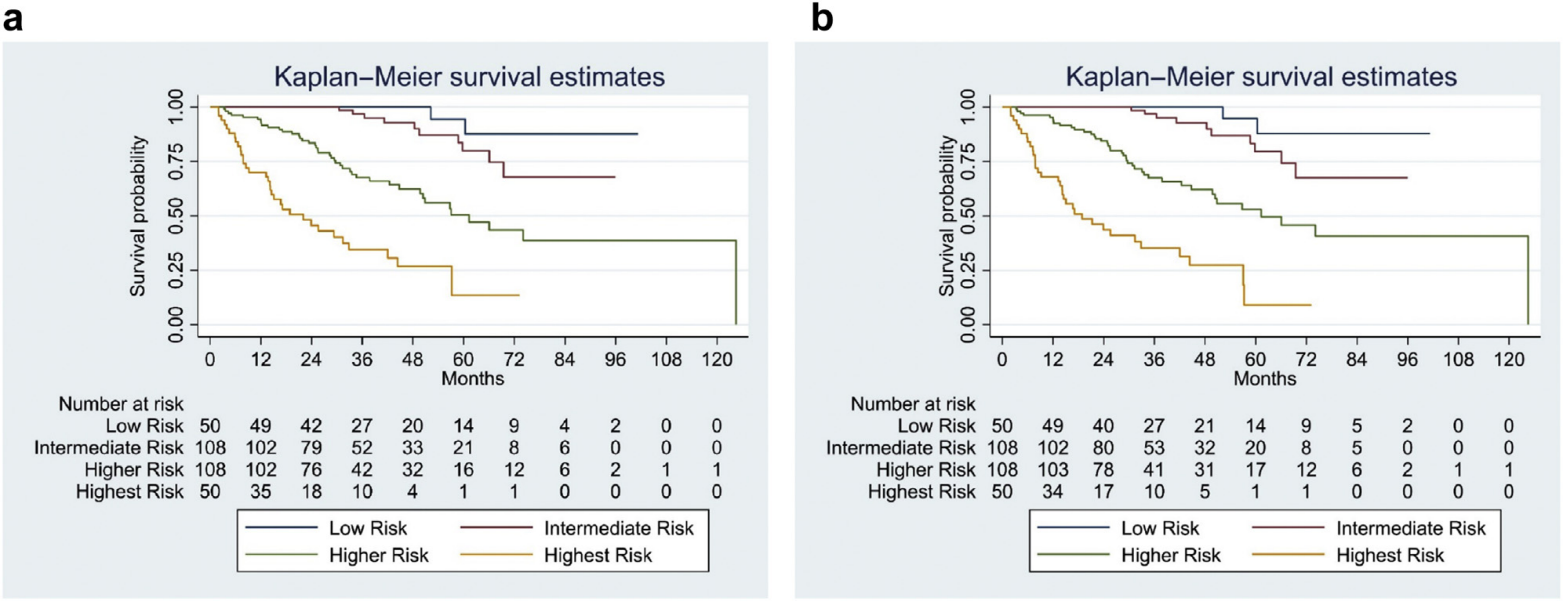
Kaplan Meier curves of the composite primary outcome (≥50% decline in eGFR or progression to end stage kidney failure) observed in the full model (a) with race and (b) without race: the well separated curves indicate good discriminant ability of both the models. eGFR, estimated glomerular filtration rate.

Kaplan–Meier curves between risk subgroups (Figure 3a and b) demonstrate well-separated survival curves for each risk group using both models which also reflects good discriminant function. Hazard ratios (Table 3) suggest that both models were less successful in distinguishing between low and intermediate risk groups and better at discriminating higher and highest risk groups from the low and intermediate groups.

**Table 3:** Association of risk group with composite outcome

Risk group	Events, *n* (%)	Hazard ratio (95% CI)	*P* value
Full model with race			
Low risk	2/50 (4)	1	
Intermediate risk	10/108 (9.3)	2.77 (0.61–12.65)	0.189
Higher risk	42/108 (38.9)	12.47 (3.01–51.62)	<0.001
Highest risk	33/50 (66)	41.15 (9.79–173.01)	<0.001
*P* value for trend	<0.0001		
Full model without race			
Low risk	2/50 (4)	1	
Intermediate risk	10/108 (9.3)	2.77 (0.61–12.65)	0.189
Higher risk	41/108 (37.9)	12.01 (2.90–49.77)	0.001
Highest risk	34/50 (68.0)	42.14 (10.04–176.88)	<0.001
*P* value for trend	<0.0001		

Risk stratification was based on percentiles of the linear predictor (low risk: <16th, intermediate risk: 16th to 50th, higher risk: 50th–84th, highest risk: >84th).

### Calibration

**Figure 4:**
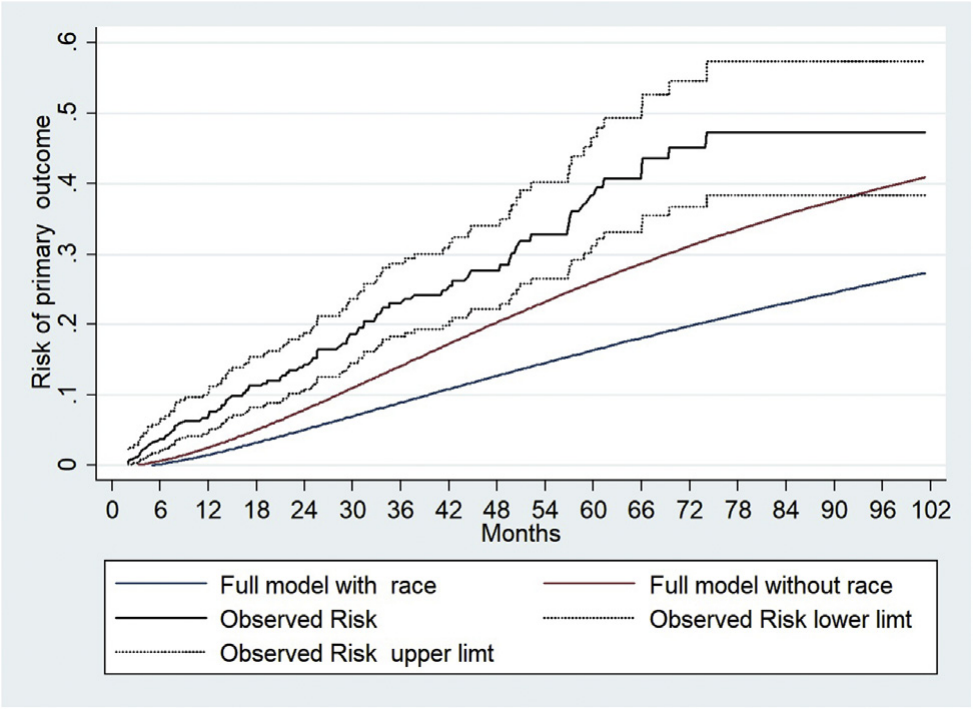
Comparison of the observed and predicted risks with both models in the entire cohort. Predicted risks are mean predicted risk and shown as red (model without race) and blue (model with race) solid lines. The observed risks were obtained by Kaplan–Meier method (black solid line) and shown with 95% CIs (black dotted line).

**Figure 5:**
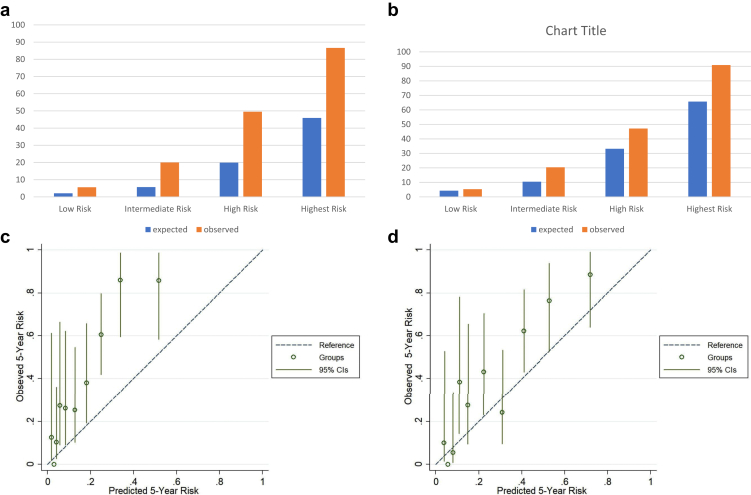
Comparison of observed and predicted risks at 5 years according to risk groups in full model (a) with and (b) without race and plotted by tenths of predicted risk using full model (c) with and (d) without race. The dashed line indicated perfect calibration, that is, the predicted and observed risks are exactly the same. The vertical lines in the observed groups denote 95% CI.

Both the models underestimated the rate of progression compared with what was observed. Figure 4 shows the mean predicted risk of progression in the follow-up period compared with the observed risk obtained by Kaplan–Meier analysis. It is evident that though overall both models underestimated the risk of progression in our cohort, it was more prominent with the model with race. Both full models with and without race underestimated the risk of reaching the primary outcome throughout the observed period for each risk group (Supplementary Figure S1A and B). Figure 5 a–d and Supplementary Figure S2 A–D show the calibration of observed and predicted risks at 5 years and 2.8 years in different risk groups and according to the tenths of the predicted risk. Both models underestimated this risk of progression in these patients showing poor calibration at 2.8 and 5 years. When we compared the observed and predicted risks in different groups at 2.8 and 5 years, both models showed a slight underestimation in the low-risk group which became more prominent with successive increase in the risk stratum (Supplementary Figure S2 A and B, Figure 5 a and b). This was more prominent in the model with race compared with the model without race.

## Discussion

The IIgANN prediction tool predicts the risk of 50% decline in kidney function or progression to end-stage renal failure in patients with IgAN at the time of kidney biopsy.^12^ It was derived and validated in a large multicentric, multiethnic cohort of 3927 patients and consists of 2 models, one with and the other without race. It requires clinical and histologic parameters easily available at diagnosis. Race/ethnicity is classified as Caucasian/Chinese/Japanese/Other. It has subsequently been validated in a large and more contemporary Chinese cohort^15^ and an Asian-Caucasian cohort,^16^ though it did not perform well in Korean patients.^17^ The original derivation and validation cohorts did not include any patients of Indian origin or from the Indian subcontinent who are known to have an aggressive disease phenotype.^4^^,^^5^ We evaluated the performance of this prediction tool in a cohort of adult Indian patients with biopsy-proven primary IgAN. This is necessary before it can be used in clinical practice in Indian patients. We first assessed the performance of the model in stratifying patients to different risk groups (low, intermediate, high, and highest risk). This discrimination ability depends on the spectrum of disease in the cohort used for validation especially in diseases such as IgAN which show significant heterogeneity in clinical presentation. The C-statistic with both models was approximately 0.845, which is similar to the original cohorts (0.81 and 0.82) and other studies^15^^,^^16^ suggesting both the models perform reasonably well in stratifying patients to different risk groups. The R^2^d for the full models with and without race was 49.9% and 44.7%, respectively, suggesting a reasonable fit. They were more effective in discriminating the high and higher risk groups than the intermediate risk from the low-risk group as seen in Table 3. Kaplan–Meier analysis showed well-separated survival curves in the risk groups stratified based on these models. Thus, patients in highest and high-risk groups had poorer survival than the intermediate and low-risk groups indicating the model could identify patients at high risk of progression at the time of kidney biopsy. This also suggests that our cohort had adequate representation of patients from different risk groups. In a Chinese cohort,^15^ the full model with race performed better with significant improvement in risk reclassification to predict the 5-year risk. However, in another study by Zhang *et al.*^16^ in a large Chinese-Caucasian cohort (8.3% Caucasian patients) of 1275 patients, there was good calibration for the full model without race, but it overestimated the risk over 3 years when race was included. Compared with our patients, this cohort had less severe disease with higher median eGFR (82.8 ml/min per 1.73 m^2^), lower proteinuria (1.2 g/d), and higher historical use of renin-angiotensin-aldosterone blockers (>75%) with a longer follow-up (3.8 years). The tool did not perform well in predicting the rate of progression to the primary outcome in our patients with suboptimal calibration. We observed only marginal improvement in risk reclassification with the full model with race compared with the full model without race at 2.8 and 5 years probably because the original derivation cohort did not include patients of Indian ethnicity. Both the models, with and without race, underestimated risk of progression when compared with the observed outcomes in the cohort at 2.8 and 5 years. This was evident in all 4 risk groups (Figure 5), but the gradient increased with progressive increase in risk. This was more prominent with the model with race compared with the model without race at 2.8 years. The 3-year outcomes of the prospective GRACE-IgANI cohort from south India also suggest that Indian patients have poorer renal survival.^5^ Although it was not a validation study, the authors demonstrated that the IIgANN tool underestimated the risk of composite outcomes in their patients, and this was more evident in the higher risk groups. The area under the curve of the ROC curve was 0.8135, though it has not been specified which model was used for risk prediction.

Thus, though the IIgANN model accurately distinguishes severity of the disease at presentation (i.e., low, intermediate, high, or highest risk) in Indian patients, it underestimates the trajectory of progression overall and also within each group. This may actually reflect the more aggressive disease phenotype in Indians; even patients who have low and intermediate risks seem to progress faster than what is anticipated in Western and even Chinese populations. Despite our patients being younger than other cohorts,^12^^,^^15^ they had lower eGFR and higher proteinuria, higher prevalence of mesangial hypercellularity, and 27.5% reached the primary outcome during a median follow-up of 2.8 years. Furthermore, 16.5% of the patients had crescents on their kidney biopsy specimens. These findings suggest that our patients have more severe disease, and it cannot be simply attributed to a delay in diagnosis.

Our study had certain limitations. This is a single-center retrospective cohort study. Though we had good baseline data (only 3.7% patients were excluded because of missing data), the follow-up may have been affected by the retrospective nature of the study. Because we are a subsidized public hospital that provides specialist care, many of our patients are socioeconomically disadvantaged and travel from distant, often rural areas and those with milder disease may discontinue follow-up especially if they have stable disease. However, we have tried to maintain follow-up with these patients telephonically and by e-mail as was feasible. We also had 27.5% patients who reached primary outcome and 65.5% of them did so by 2.8 years.

These factors may have contributed to the overall shorter median follow-up than the original cohorts which may affect the calibration results. We tried to address these lacunae by evaluating the models at 2.8 years which also produced similar results. India has a vast population with multiple ethnic groups which may differentially impact the outcome of the disease. We are a tertiary care public teaching hospital located in northern India, so our patients predominantly hail from north, west, and central India but being located in the capital city, we have patients from other parts of the country and Nepal in this cohort. It is a relatively smaller cohort and had <100 primary outcome events which is ideally required for validating prognostic models.^18^ Simulation-based approach to calculate the sample size for external validation of a prediction model taking into account the linear predictors is more precise^19^ but was not possible in this setting as this was a retrospective study limited by the number of patients seen at out center during this period.

To conclude, in a validation study, we evaluated the IIgANN prediction tool in Indian patients, an ethnic group that was not included in the original cohort and has not been studied until date. It was effective in distinguishing different risk groups of patients, but both models with and without race underestimated the trajectory of progression of kidney disease. Multicentric cohort studies with longer follow-up are required to assess the performance of these equations in Indian patients before implementing them in clinical practice. A specific coefficient may be required for the Indian race to improve the calibration of this model. We also need to consider the impact of crescents in kidney biopsy and management strategies after the diagnosis of IgAN on the disease outcome. Practice patterns are especially important considering the variability in the use and maximization of renin-angiotensin-aldosterone blockers, the threshold for starting immunosuppression, the type of immunosuppression used, and the risk of intercurrent infections, all of which are known to affect the kidney function.

## Disclosure

All the authors declared no competing interests.

## Data Sharing Statement

Data relevant to this study have been provided in the document. Additional information may be made available as required.

## Supplementary Material

Supplementary File (PDF)

**Supplementary Data.** TRIPOD checklist for validation of prediction models.

**Figure S1.** Observed and predicted survival in all risk groups using the (A) full model with race and (B) without race.

**Figure S2.** Comparison of observed and predicted risks at 2.8 years according to risk groups in full model with (A) and without race (B) and plotted by tenths of predicted risk using full model with (C) and without race (D). The dashed line indicated perfect calibration, that is, the predicted and observed risks are exactly the same. The vertical lines in the observed groups denote 95% CI.
